# A Scoping Review of ‘Smart’ Dressings for Diagnosing Surgical Site Infection: A Focus on Arthroplasty

**DOI:** 10.3390/bioengineering11101049

**Published:** 2024-10-21

**Authors:** Samuel W. King, Alexander Abouharb, Thomas Doggett, Mohamad Taufiqurrakhman, Jeya Palan, Bulut Freear, Hemant Pandit, Bernard H. van Duren

**Affiliations:** 1 Leeds Institute of Rheumatic and Musculoskeletal Medicine, University of Leeds, Chapel Allerton Hospital, Chapeltown Road, Leeds LS7 4SA, UK; 2 Leeds Teaching Hospitals NHS Trust, St. James’s University Hospital, Beckett Street, Leeds LS9 7TF, UK; 3 School of Medicine, Worsley Building, University of Leeds, Woodhouse, Leeds LS2 9JT, UK; 4 School of Medicine, Anglia Ruskin University, Chelmsford CM1 1SQ, UK

**Keywords:** smart dressing, wearable sensor, wound infection, periprosthetic joint infection, PJI, remote monitoring

## Abstract

Early diagnosis and treatment of surgical wound infection can be challenging. This is especially relevant in the management of periprosthetic joint infection: early detection is key to success and reducing morbidity, mortality and resource use. ‘Smart’ dressings have been developed to detect parameters suggestive of infection. This scoping review investigates the current status of the field, limited to devices tested in animal models and/or humans, with a focus on their application to arthroplasty. The literature was searched using MEDLINE/PubMed and Embase databases from 2000 to 2023. Original articles assessing external sensing methods for the detection of wound infection in animal models or human participants were included. Sixteen articles were eligible. The results were broadly divided by sensing method: colorimetric, electrochemical and fluorescence/photothermal responses. Six of the devices detected more than one parameter (multimodal), while the rest were unimodal. The most common parameters examined were temperature and pH. Most ‘smart’ dressings focused on diagnosing infection in chronic wounds, and none were tested in humans with wound infections. There is limited late-stage research into using dressing sensors to diagnose wound infection in post-surgical patients. Future research should explore this to enable inpatient and remote outpatient monitoring of post-operative wounds to detect wound infection.

## 1. Introduction

Surgical site infection (SSI) is the infection of wounds created by invasive surgical procedures, characterized by erythema, localized pain, wound discharge and dehiscence. Whilst SSIs are a relatively common post-operative complication, the impact of SSI occurrence on subsequent clinical outcomes varies by surgical specialty and procedure. These may be classified and described as superficial incisional, involving the skin or subcutaneous tissue layers; deep incisional, involving the deeper structures; or involving the organ or space operatively managed [1]. Post-operative wound infection is the third biggest cause of healthcare-associated infection [2]. They cost the US healthcare system over $3 billion per year and increase mortality by 2–11 times [1,3]. A total of 69% of these are diagnosed as outpatients [4] and are often overlooked or diagnosed late [5]. In arthroplasty surgery, superficial wound infection can lead to deeper periprosthetic joint infection (PJI), which is a potentially devastating surgical complication. PJI is one of the most common indications for early revision surgery [6,7]. The first 30 days are the highest risk period [8]. PJI is associated with a high rate of morbidity, and has a five-year mortality rate following infection of 25.9% [9]. Data from over 10 years ago showed up to 27% of revisions were due to infection. This is a costly complication, with over £30,000 costs per PJI knee revision in the National Health Service [10]; with inflation, this equates to approximately £40,000 today [11]. For comparison, this was triple the cost of an aseptic revision.

Diagnosis of infection remains challenging. Physical findings of warmth, erythema, pain and deranged laboratory parameters in blood tests are suggestive but are neither specific nor sensitive [12]. In the absence of a sinus tract or visible implant through the wound, invasive investigations remain the only way to diagnose PJI definitively, with no other clinical tool confirming the diagnosis with complete accuracy [12,13]. Remote monitoring of wounds following arthroplasty for early indicators of SSI and wound leakage with infective features in the early post-operative period would provide the opportunity for timely diagnosis of superficial and deep infections. This association enables the potential to intervene when treatment is more effective. The use of “smart” dressings is increasingly being identified as a method of improving the early diagnosis of SSIs. Unlike traditional dressings, “smart” dressings incorporate materials with advantageous biochemical properties to enhance healing and may also include integrated sensors to allow for real-time monitoring of surgical wounds. A range of sensing parameters have been explored, with changes in temperature, pH, uric acid and glucose all frequently evaluated owing to their ubiquity and low cost [14]. A range of monitoring methods have also been developed, allowing for the monitoring of these parameters in wearable dressings, including the use of microelectronic sensors, microprocessors and optical (colorimetric and fluorometric) and electrochemical sensors.

A number of reviews describe progress in the monitoring of healing and diagnosis of infection in chronic wounds [15,16,17], but many of the studies presented have not yet progressed beyond in vitro testing [18,19,20]. Meanwhile, the use of smart dressings to improve post-operative wound monitoring is an area of increasing interest, but most of the literature focuses on the management of chronic wounds rather than acute surgical wounds. Additionally, the current state of products tested ex vivo, in vivo and in clinical practice has not recently been reviewed. It follows that a need for a review of the existing literature is evident to identify and evaluate the range of novel biomaterials, sensing parameters and sensing methods in use in current “smart” dressing technologies.

This scoping review aims to evaluate the current literature to identify the range of materials, parameters and methods being used in animal models and humans. This is with the goal to identify products most likely to have potential for application as wound dressing sensors to detect infection in post-operative patients. If studies are available that focus on acute surgical wounds, this will be further examined to establish the current state of products and their future potential.

## 2. Materials and Methods

A scoping review of the literature was undertaken. A pre-determined search strategy was used. MEDLINE/PubMed and Embase were searched from 1 January 2000 to 11 December 2023 for potential eligible studies. Search terms relating to sensors and smart and wearable technology were combined with those relating to infection, which were in turn combined with those relating to wounds, incisions and injuries. A full description of this strategy is available in Table 1. De-duplication of the results was performed using a strategy described by Bramer et al. [21].

Studies were eligible for inclusion if they were published between 1 January 2000 and 11 December 2023 with a full text available that was written or translated into English. Other mandatory inclusion criteria were as follows: assessment of the use of external sensing methods to detect and/or diagnose wound infection; and use in ex vivo or in vivo animal models of wound infection or wound infection in human participants. In vitro studies, conference abstracts, trial protocols and those that did not fulfil the inclusion criteria were excluded. Review articles were excluded, but their reference lists were searched for additional studies eligible for inclusion. Trial protocols and conferences were used to search for full-text published results.

**Figure 1:**
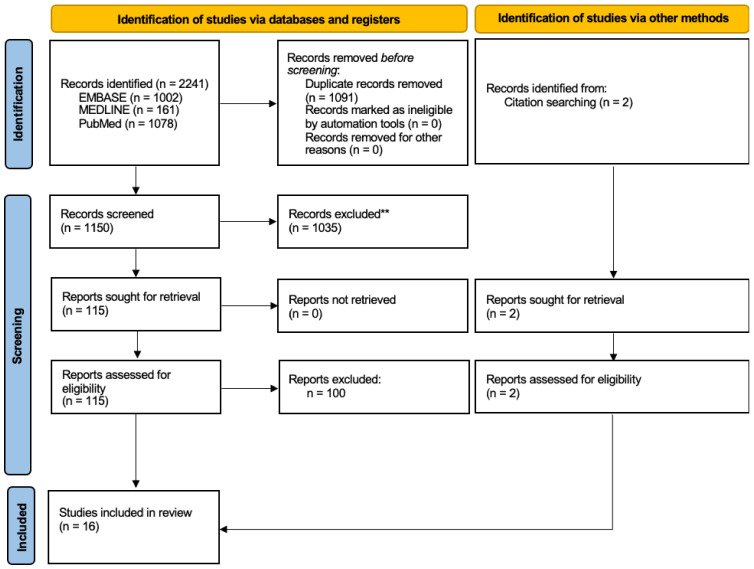
PRISMA flow diagram illustrating the results of the search and review strategy.

Two authors (AA and TD) reviewed the results of this search, with the first author (SWK) providing input to arbitrate any disagreement. The senior author (HGP) was available to provide input when required. A title/abstract review was performed first, followed by a full-text review. A PRISMA [22] flow diagram demonstrating the results of the review is shown in Figure 1.

## 3. Results

**Figure 2:**
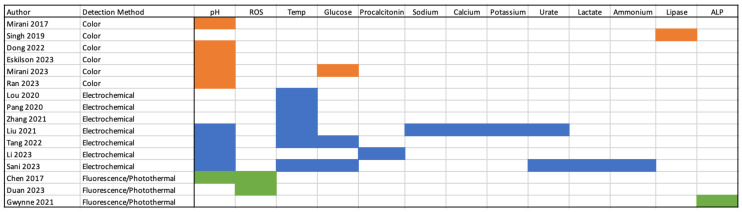
Breakdown of studies by parameter assessed and class of method utilized [23,24,25,26,27,28,29,30,31,32,33,34,35,36,37,38].

**Figure 3:**
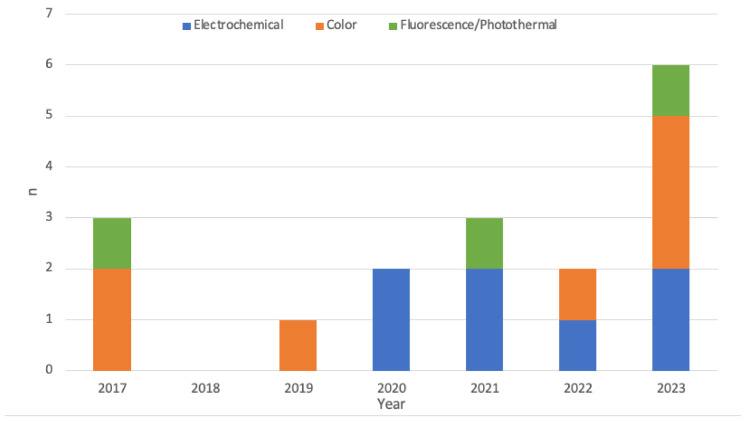
Publication years of studies included and broken down by detection method.

Sixteen manuscripts were eligible for inclusion. These articles were classified by sensing method, which were broadly characterized as colorimetric, electrochemical and fluorescence/photothermal responses. Ten articles detected a single parameter used for the detection of infection while six were multimodal. There were no studies in human participants. The most common parameters examined were temperature and pH. A breakdown of the frequency of parameters examined is available in Figure 2. The literature was searched from 2000, but 13 of the 16 included studies are from the current decade and none were published before 2017 (Figure 3).

### 3.1. Colorimetric

**Figure 4:**
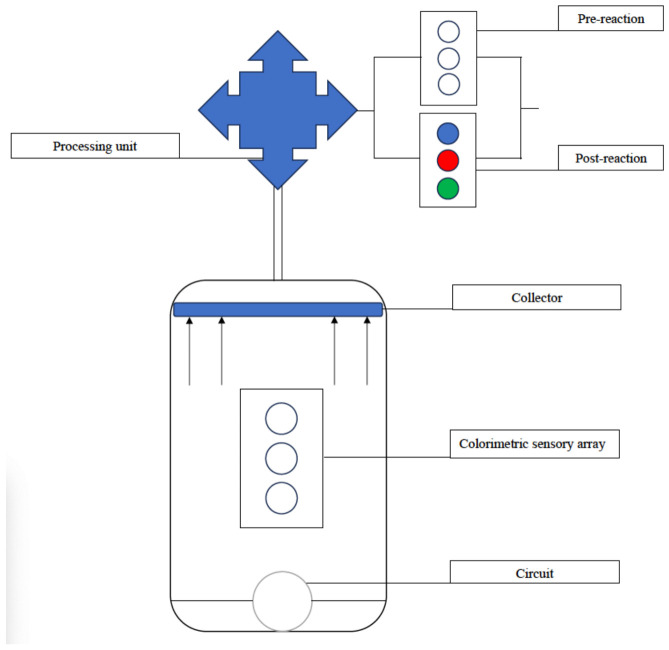
Schematic representation of conversion of colorimetric sensor output into electronic data.

Color changes in dressing sensors can be analyzed with photographic software or the naked eye, depending on the product [23]. They require no power source and are generally less resource-intensive to produce; however, real-time monitoring requires constant observation and challenges include the calibration of color changes and leaching of indicator dyes. Color changes may also be converted into electrical signals for more objective interpretation. This is performed in a process schematically represented in Figure 4.

#### 3.1.1. Unimodal

The pH of intact skin is slightly acidic at approximately 4–6, and while an acute wound exposes the neutral (pH 7.4) underlying tissue, inflammatory activity soon reduces this back to 4–6 as a mechanism for discouraging pathogenic growth [14,39], while chronic and infected wounds are closer to 7–9 [24,39,40]. An increase in pH often precedes clinical symptoms, and so is a common parameter monitored for wound infection [41]. Colorimetric methods mostly monitored pH, which was likely due to the ready availability of pH indicators that change color within the visual spectrum.

A hydrogel-based wound dressing made from bacterial nanocellulose was reported. A pH-responsive dye was encapsulated within mesoporous silica nanoparticles in an attempt to prevent dye leakage [24]. This dressing was applied to infected and non-infected porcine wound models; the former was created by inoculation with *Staphylococcus aureus* (*S. aureus*). Dressings applied to non-infected wounds demonstrated a yellow-green color, indicating physiological pH, while those applied to infected wounds displayed a yellow to deep blue transition in under a minute, suggesting a pH of approximately 8. The difference was easily discriminated with the naked eye.

Another hydrogel dressing was produced that included phenol red to demonstrate the pH of infected wounds [23]. *S. aureus*-infected abscess models in mice were used to validate this. The pH was calculated using color images captured and processed with a smartphone application. Color changes seen with infection demonstrated a pH decrease to 5.6 on initial infection, up to 7.2 within 2 days to demonstrate infection, and a decrease back to normal skin pH (6.5) over 4 days.

A film-based band aid was assessed by Dong et al. [25]. This consisted of Bi2S3 nanoflowers loaded with rhodium nanoparticles and bromothymol blue in an LB agar film. They were applied to *S. aureus*-infected wound models in mice, with a color change of blue to yellow to indicate the presence of *S. aureus*. NIR light was used to induce a photothermal response as part of the treatment of these infected wounds

A full evaluation of “theranostic” systems is out of the scope of this review but must also be mentioned. The general principle of theranosis is combining diagnosis with treatment [42]. Within the field of wound infection, this is relevant in the context of topical antimicrobial delivery. Over-treatment in non-infected wounds contributes to the development of antimicrobial resistance. The capacity to accurately diagnose infection and deliver antimicrobials from within the wound dressing system promises to combine the advantages of early treatment whilst avoiding the danger of over-treatment.

A theranostic dressing was produced by Singh et al., consisting of a polyurethane scaffolding loaded with a ciprofloxacin-based prodrug (Pro-Cip) and a chromogenic probe for lipase [26]. This detects lipase in some lipase-secreting bacteria, e.g., *P. aeruginosa*. A full-thickness skin burn on ex vivo pig skin was inoculated with *P. aeruginosa*, resulting in a color change from yellow to dark green. Additionally, the presence of lipase resulted in the cleavage of ester linkages in Pro-Cip and the release of antibiotics.

GelDerm (patented by 4M Biotech, Victoria, BC, Canada) is a dressing that uses an array of porous sensors consisting of mesoporous resin beads doped with a pH-responsive dye embedded in a 3D-printed alginate dressing [27]. In a study evaluating this device, image processing software with a phone application was used to detect color change, with brilliant yellow and cabbage juice as indicators. Ex vivo testing was undertaken in pig skin inoculated with *Pseudomonas aeruginosa* (*P. aeruginosa*) and color change was viewed by the naked eye and image processing. A clear color change was observed in colonized skin, with a dose–response curve based on the quantity of bacteria calculated using the smartphone application.

#### 3.1.2. Multimodal

The same group recently published on a developed version of GelDerm capable of mapping bacterial infection by a colorimetric pH sensor [28]. Glucose sensors were also incorporated for a diabetic population and utilized glucose oxidase to ultimately oxidize iodine, causing a light yellow to dark red color change. In murine wound infection models with *Escherichia coli* (*E. coli*), the pH was significantly higher compared with non-infected wounds, which was consistent with independent pH probe testing. Treatment with antibiotics reduced this pH in a manner consistent with infection resolution.

### 3.2. Electrochemical

**Figure 5:**
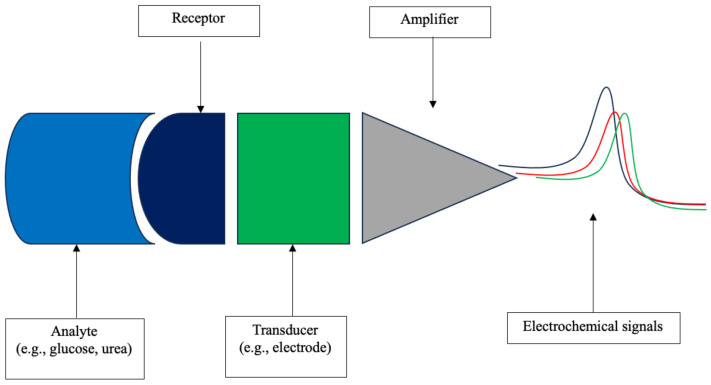
Detection of chemical analyte and its conversion to electronic data.

Electrochemical measurement of parameters was most often conducted in conjunction with wireless Bluetooth communication of information to an electronic device. This allows for the real-time transfer of information with a greater ability to analyze trends of parameter changes. It is also more feasible to measure multiple parameters, and several of the devices described were multimodal. However, there is a concomitant increase in costs and challenges associated with data transfer and device powering. Figure 5 schematically demonstrates the detection of analyte and its conversion to electronic data.

#### 3.2.1. Unimodal

Local temperature increase is an early predictor of wound infection [43,44]. Wound temperature is often raised compared with the surrounding skin before other more obvious symptoms of wound infection, such as redness, swelling or odor, are present [45,46]. It is, therefore, a popular target for monitoring wound status.

Lou et al. report a double-layer dressing with a flexible temperature-sensing external layer with a power manager, Bluetooth transmitter and data processing circuit, and a lower layer consisting of collagen–chitosan [29]. This communicated with a smartphone application. Pig models with incisional wounds were inoculated with *S. aureus*. Rectal temperature is expected to be higher than skin temperature given the cooling effects of the ambient environment. In control experiments, the group found this to be approximately 1–1.5 °C below rectal temperature in a 24 °C environment. Early wound infection models were inoculated at the time of wound creation, and wound temperature peaked quickly to 39 °C, which was 0.5 °C above rectal temperature. Late infection (inoculation at day 14 after wounding) exhibited a gradual increase, starting below rectal temperature and exceeding it approximately 12 h after inoculation. Early and late models of infection using wound temperature provided a warning of infection at 30 and 20 h before rectal temperature, respectively.

The same group developed a wound dressing that also incorporated antimicrobial treatment [30]. There was an upper layer of polydimethylsiloxane encapsulating flexible electronics, temperature sensors and ultraviolet-light-emitting diodes (UV-LEDs), and a lower UV–responsive antibacterial hydrogel layer with gentamicin. Bluetooth was used to transmit data. When the local temperature reached >40 °C, an infection alarm was triggered. Treatment was started by activating UV-LEDs, which in turn released antibiotics from the UV–responsive hydrogel. A pig model of an infected wound with *S. aureus* was used. Early and late (14 days post-injury) models of wound infection both demonstrated a warning of infection prior to pathological increases in rectal temperature measurement.

Zhang et al. developed a flexible integrated sensing platform with a flexible sensing chip for temperature, a printed circuit board and a customised smartphone application with Bluetooth communication [31]. Rabbit back wounds were infected with *S. aureus*, *P. aeruginosa* and *K. pneumoniae*, and the temperature was updated every 2–10 s in real time. In the uninfected, temperature increased for first the 3–4 days during hyperaemia and inflammation, plateauing at this point. Bacteria were added to some of the wounds on the third day. Local temperature increased from 37 to 38.5 °C in *S. aureus* infection, while responses to *P. aeruginosa* and *K. pneumoniae* were less marked. An increase in rectal temperature on day two after inoculation or day two for wound temperature was predictive of a risk of infection, suggesting direct measurement of local temperature gave an earlier warning for infection.

#### 3.2.2. Multimodal

A multifunctional wound dressing was designed with a sensing layer sandwiched within a self-healing elastomer, which was able to monitor glucose, pH and temperature [32]. It utilized a dual-electrode system for glucose (Prussian blue/glucose oxidase) and pH (polyaniline). In mouse models of infected versus uninfected wounds, pH and temperature were higher in the infected models by day 2–3 following inoculation, while glucose was not as discriminating.

Wound infection may lead to sepsis, which is a leading cause of morbidity and mortality globally [47]. Procalcitonin is a key biomarker, rising sharply during a septic state [48]. Li et al. developed a wearable battery-free wound dressing sensor with a microfluidic module, a wound sensing module for pH and procalcitonin and a near-field communication module and temperature sensor embedded within a flexible printed circuit board [33]. The unit was encapsulated within a thin layer of Ecoflex (Smooth-On Inc., Macungie, PA, USA). Data transmission and power supply were wireless with a smartphone that activated the circuit. The microfluidic system collected and filtered the wound exudate. Rats were used as wound models, with dorsal circular incisions. Infection was simulated by intra-peritoneal injection of lipopolysaccharide versus a control of PBS. No bacteria were introduced into the wound. A significant increase in temperature was detected by the sixth hour in the sepsis model compared with controls. Procalcitonin and pH were also significantly higher by the second hour.

Uric acid cannot be metabolized endogenously in mammals but is metabolized by bacteria containing uricase such as *S. aureus* and *P. aeruginosa* [14]. A group developed a multiplex-sensing bandage system for real-time monitoring of sodium, potassium and calcium ions, urate, pH and temperature [34]. They used a graphene-based thermally responsive resistor for temperature, and electrochemical detection of the ions. These formed a multi-sensor array in conjunction with a flexible circuit board. An external power supply was required, and information was transmitted via Bluetooth. Rat wound models were created and then inoculated with *S. aureus*. Compared with controls, the infected wound demonstrated progressively more alkaline conditions, an increase in uric acid and a temperature difference of around 1.5 °C in the infected wound by around 20 h. There was a significantly lower concentration of calcium ions in infected wounds, while sodium and potassium ions were less discriminating.

A stretchable wearable dressing was designed with sensors for temperature, pH, ammonium, glucose, lactate and uric acid using electrochemical biosensor arrays [35]. Zucker diabetic fatty rats were used as a model for diabetic infected wounds with a mixture of MRSA and *P. aeruginosa*. Uric acid, lactate, ammonium, pH and temperature were detected and increased daily from baseline in infected models compared with minimal change after wounding in non-infected models. These all returned toward baseline following antimicrobial treatment.

### 3.3. Fluorescence/Photothermal

**Figure 6:**
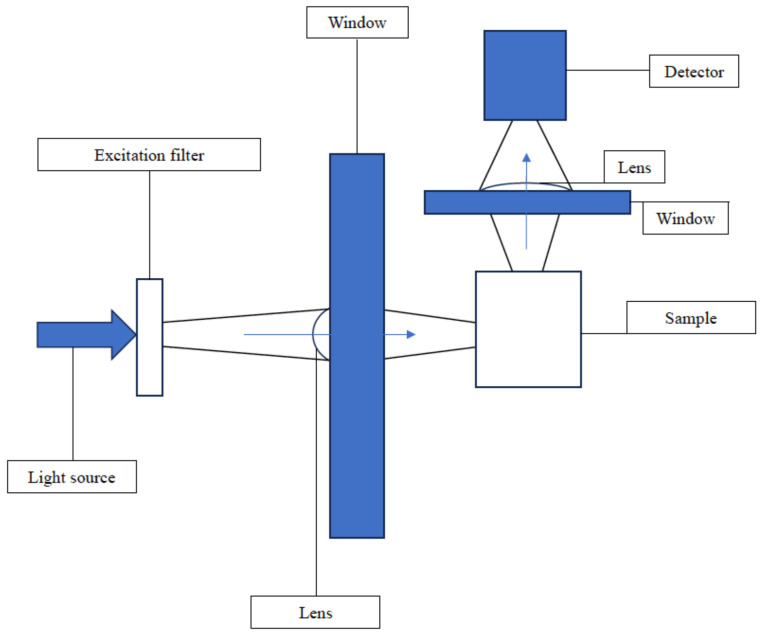
Schematic illustration of a fluorescence sensor.

Substances may be detected using a fluorescence or photothermal reaction, whereby exposure of a signaling substance to a specific wavelength of light produces a corresponding light signal that can be detected by the naked eye and/or with an electronic sensor. The latter process is demonstrated in Figure 6.

#### 3.3.1. Unimodal

Reactive oxygen species were detected with a smart dressing evaluated by Duan et al. [36]. IR820 exhibits photothermal conversion in response to near-infrared (NIR) light. When a near-infrared (NIR)-active dye (IR820) was trapped in calcium-ion sealed porous silicon-based carriers, a superior photothermal response was observed. These carriers were embedded within a polyurethane membrane and decay in response to reactive oxygen species (ROS). The resulting reduced photothermal signal from free released IR820 was detectable with a smartphone thermal camera. By applying the wound dressing to diabetic mouse wound models inoculated with *S. aureus*, the presence of ROS could be detected. However, a control group without infection was not utilized.

Gwynne et al. used alkaline phosphatase (ALP) as a biomarker for bacterial infection [37]. It is responsible for the hydrolysis of phosphoesters to release the phosphate required for bacterial cell growth. It is present in *E. coli, P. aeruginosa* and *S. aureus*. The authors used an ALP-responsive colorimetric and fluorescent probe. The probe was encapsulated in polyvinyl alcohol-based hydrogels. This displayed a yellow to purple color change in fluorescence after 24 h of incubation in ex vivo *S. aureus* porcine skin wound models, with the difference easily visible with the naked eye.

#### 3.3.2. Multimodal

Chen et al. devised a smart bandage with luminescent porous silicon particles loaded with ciprofloxacin sandwiched between a polyurethane membrane and chitosan film [38]. These responded to increases in pH and the presence of ROS, which are both features of infected wounds. Exposure to oxidant species oxidized the red luminescent surface of the particles, releasing blue luminescent ciprofloxacin, which was visible to the naked eye or smartphone camera. They compared responses in surgically created wounds in mice, comparing *S. aureus* inoculation versus uninfected acute wounds. The pH in the infected wounds increased to 7.9, while the uninfected remained at approximately 7.5. There was a significant difference in luminescence (bluer in the infected model) by day two following application, which was maintained to day four, i.e., the end of the observation period. Under UV light, a difference in color was visible to the naked eye and could also be interpreted by a phone camera.

## 4. Discussion

At the time of searching, there were no in-human studies of wound infection diagnosis using wearable sensors in a dressing, although some of the devices had been tested in healthy human participants for durability and tolerance, or with in vitro testing of wound exudates. Most of the studies focused on the diagnosis of infection in chronic wounds. There were inherent limitations to the methodology used in the included studies. The lack of clinical studies means caution must be exercised when interpreting results.

Many of the included studies included the application of relatively large numbers of bacteria, given they were intended to mimic the environment of a non-sterile chronic wound. Surgical wounds are much less likely to be grossly contaminated. Additionally, while many of the animal model wounds were created in a manner similar to a surgical wound, only one study reported closure of the wound [32]. There were no studies of orthopedic surgery or models of orthopedic wounds. Finally, this review was purposefully limited in scope to identify wound dressing sensors at a more advanced stage of development, and searched only biomedical journal databases. Sensors in earlier stages of development and published in journals in other fields are, therefore, excluded from this review, introducing a potential bias in the review process.

It is apparent that the field of wound infection dressing sensors is, therefore, in its relative infancy and has not progressed past the in vitro stage of development. There is additionally a gap in the literature with respect to wound dressing sensors for the diagnosis of wound infection in post-surgical patients.

The included studies are broadly categorized by type of sensing method utilized: colorimetric, electrochemical and fluorescence/photothermal responses. Each method has its limitations. Colorimetric sensors may be less resource-intensive to produce and can often be interpreted by the naked eye, negating the requirement for technological input. However, data resolution is minimal; they are often only able to undergo a color change once; and remote monitoring potential is limited unless they are used in conjunction with further technology. Electrochemical sensing can provide detailed real-time information about the local environment of a wound. However, this may be costly, especially to produce sensors for individual analytes and when used to detect changes in multiple parameters. Additionally, a greater degree of data processing, data storage and/or power supply may be required, further increasing cost and size. Finally, fluorescence/photothermal response sensors may be used as “theranostic” sensors, i.e., simultaneous diagnosis and treatment. Here, the application of light will both produce a visible signal for the diagnosis of infection and cause the release of antimicrobial agents for its treatment. However, development costs are high, and the field of theranostic wound dressings remains experimental.

There is a growing interest in wound monitoring for the early detection of infection. The “Blackbox” nature of healing and infection under traditional dressings [29] and the potentially devastating consequences of a wound infection make an objective early warning system an extremely attractive prospect. At present, the UK’s National Institute for Healthcare and Clinical Excellence and the USA’s Centers for Disease Control and Prevention both provide guidance around the prevention and treatment of surgical site infections but not around their routine surveillance in terms of the frequency or nature of assessment. Smart dressings for wound infection would allow constant monitoring and also data on the presentation and features of early surgical site infection to assist further guidelines.

A wound dressing sensor for use in post-operative patients would allow the detection of a suspected infection without removal of the dressing itself. This could also be remotely monitored by a clinical team. This would allow monitoring without additional resource-intensive follow-up appointments and provide peace of mind for surgical patients in the community. Patients with wounds demonstrating a higher likelihood of infection can be further assessed by a surgical team.

Overuse of antibiotics in healthcare is a major global concern. The development of antibiotic-resistant bacteria is accelerated by indiscriminate use of broad-spectrum agents. Smart dressings could provide an opportunity for the early detection of infection to limit antibiotic use to appropriate, targeted and timely therapy.

The use of these solutions in clinical practice would have to be contextualized. The diagnostic reliability of any dressing sensor would need to be formally quantified. In addition, infrastructure around monitoring information from wound dressing sensors for patients in the community would have to be developed. Development of a clear protocol would be key to ensuring that responsibility and liability around data from dressing sensors was acted upon in an appropriate and timely manner.

Future studies should focus on the development of wound dressing sensors that allow inpatient and remote outpatient monitoring of post-operative wounds to allow the early detection of wound infection in this patient cohort. The most promising parameters appear to be pH and temperature; these are the best understood and most frequently studied parameters. They have both demonstrated the detection of infection in animal models, and sensors to detect changes in pH and temperature are readily available, accurate and relatively inexpensive to produce. Additionally, moisture detection would be informative given the strong association of infection in the context of wound leakage for surgical wounds.

## 5. Conclusions

Wound dressing sensors have been studied in animal models with some success and aim to diagnose infection using a range of parameters. They are in their relatively early stages of development and have not yet been tested in humans. They have the potential to revolutionize the remote monitoring of post-operative patients in the community but require further study to understand their possible applications and the infrastructure required to support their use in a clinical context.

## Figures and Tables

**Table 1:** Example of search strategy as utilized in OVID EMBASE.

1 (sensor or biosens* or smart or wearable or detector).ab. 398767 2 (infect* or septic* or sepsis or purulen* or pji or cellulit*).ab. 3601356 3 (wound or incision or incised or injur* or operat* or scar*).ab. 4711934 4 1 and 2 and 3 1501 5 remove duplicates from 4 1206

## Data Availability

The raw data supporting the conclusions of this article will be made available by the authors on request.
